# Chronic inflammation contributes to the development of hepatocellular carcinoma by decreasing miR-122 levels

**DOI:** 10.18632/oncotarget.7740

**Published:** 2016-02-26

**Authors:** Changfei Li, Mengmeng Deng, Jun Hu, Xin Li, Lizhao Chen, Ying Ju, Junli Hao, Songdong Meng

**Affiliations:** ^1^ CAS Key Laboratory of Pathogenic Microbiology and Immunology, Institute of Microbiology, Chinese Academy of Sciences (CAS), Beijing, China; ^2^ School of Biomedical Sciences, Chengdu Medical college, Chengdu, Sichuan, China

**Keywords:** C/EBPα, miR-122, IL-6, c-myc, TNF-α

## Abstract

Persistent inflammation in chronic hepatitis plays a major role in the development of hepatocellular carcinoma (HCC). In this study, the major inflammatory cytokines expressed in chronic hepatitis, IL-6 and TNF-α, induced a marked decrease in microRNA-122 (miR-122) levels, and miR-122 expression was downregulated in the livers of chronic hepatitis B (CHB) patients. The decrease of miR-122 caused upregulation of the proinflammatory chemokine CCL2. IL-6 and TNF-α suppressed miR-122 both by directly downregulating the transcription factor C/EBPα and indirectly upregulating c-myc, which blocks C/EBPα-mediated miR-122 transcription. In addition, IL-6 and TNF-α levels were elevated and miR-122 levels were decreased in mouse and rat models of diethylnitrosamine (DEN)-induced HCC. Restoration of miR-122 levels through delivery of agomir-122 suppressed DEN-induced hepatocarcinogenesis in mice. Our results show that inflammation-induced miR-122 downregulation in hepatitis contributes to carcinogenesis and suggest that increasing miR-122 may be an effective strategy for preventing HCC development in CHB patients.

## INTRODUCTION

MicroRNAs (miRNAs) are a class of small noncoding RNAs of approximately 22 nt in length that often inhibit gene expression at the posttranscriptional level by binding to mRNAs, often in the 3′-UTR. miRNAs may regulate approximately half of the human transcriptome, leading to the degradation and translational repression of target mRNAs [1]. As the most abundant liver-specific miRNA, miR-122 accounts for about 70% of the total miRNA population in the adult liver. miR-122 is involved in various physiological and pathological processes in the liver, such as liver development, lipid metabolism, stress responses, and viral infections [2].

There is growing evidence that miR-122 is important in the development and metastasis of hepatocellular carcinoma (HCC). Decreased miR-122 expression and increased expression of miR-122 target genes are observed in HCC tumors compared to nontumor tissues, and loss of miR-122 expression is associated with hepatocarcinogenesis, metastasis, poor prognosis, and reduced response to chemotherapy [3, 4]. In addition, miR-122 knockout (KO) mice develop hepatitis, fibrosis, and HCC. Importantly, restoration of miR-122 strongly inhibits tumorigenesis and reduces tumor incidence in miR-122 KO mice, indicating that miR-122 acts as an HCC tumor suppressor [5, 6]. Multiple genes targeted by miR-122 are involved in hepatocarcinogenesis, including the oncogenes cyclin G1, a disintegrin and metalloprotease family 10 (ADAM10), serum response factor (SRF), insulin-like growth factor 1 receptor (Igf1R), Wnt1, RhoA, pituitary tumor-transforming gene 1 (PTTG1) binding factor (PBF), and AKT3, as well as the glycolytic gene pyruvate kinase M2 (PKM2) [2, 3, 7–10].

Numerous preclinical and clinical studies demonstrate the pathogenic role of chronic inflammation in HCC [11–13, 14]. In chronic hepatitis, pro-tumorigenic inflammation, which is characterized by liver-infiltrating Th2 cells, regulatory T cells (Tregs), and M2 macrophages, as well as TNF-α, IL6, IL-1α and IL-1β expression, may induce persistent hepatocyte generation and survival, increasing the neoplastic transformation of hepatocytes [15–17]. Several inflammation-related signaling pathways are involved in hepatocarcinogenesis, including the NF-κB, JAK-STAT, Raf/MAPK/ERK, Wnt-β-catenin, IRAK-1, and PI3K/AKT/mTOR pathways. These pathways have many functions and involve reciprocal crosstalk, and uncovering specific targets may assist in developing more efficient liver cancer treatments.

Our previous studies show that miR-122 is downregulated in chronic hepatitis B (CHB) and HCC, and upregulation of its target PBF promotes HCC growth and invasion [10, 18]. Although chronic inflammation causes metachronous multicentric hepatocarcinogenesis, it is still unclear if inflammation affects miR-122 expression. Therefore, in this study we investigated the possible mechanisms underlying miR-122 downregulation during liver inflammation.

## RESULTS

### Inflammatory cytokines suppress miR-122 in CHB

**Figure 1:**
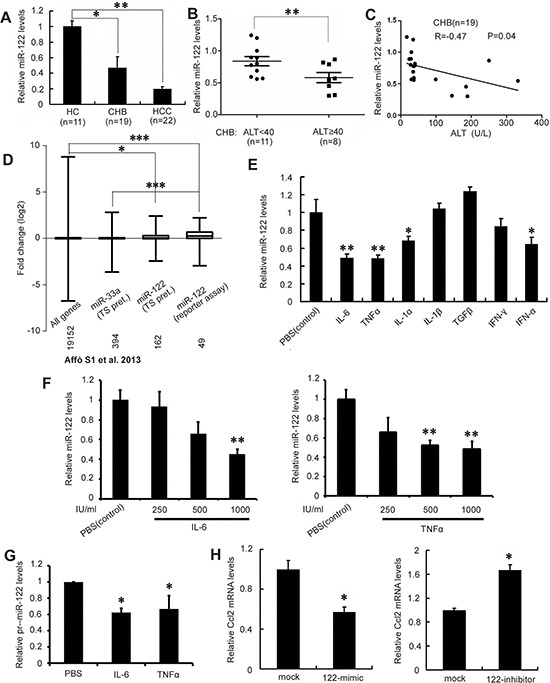
Major inflammatory cytokines in CHB suppress miR-122 **A.** Analysis of miR-122 expression in liver biopsy specimens from CHB patients, HCC tumors, and healthy controls (HC) by real-time PCR. Results were normalized to a U6 endogenous control, RNU6B. miR-122 levels in HC were arbitrarily set to 1.0. **B.** Real-time PCR analysis of miR-122 levels in CHB patients with ALT below and above 40 U/L. **C.** Correlation analysis of miR-122 levels and serum ALT levels in CHB patients. **D.** Meta-analysis of published gene expression microarray data comparing 15 alcohol-induced hepatitis samples to seven healthy livers. miR-33a and miR-122 targets predicted by TargetScan (TS) and miR-122 targets validated by reporter assay (mirbase) were analyzed. The mean fold change in expression of miR-122 targets was compared to miR-33a targets or all genes; the number of genes in each group is shown. **E.** Huh-7 cells were treated with 1000 IU/ml IL-6, TNF-α, IL-1α, IL-1β, TGF-β, IFN-γ, IFNα, or PBS as a control for 12 h. miR-122 expression was detected by real-time PCR. **F.** miR-122 levels were analyzed after treatment with the indicated amounts of IL-6 (left) or TNF-α (right). **G.** Huh-7 cells were treated with 1000 IU/ml IL-6 or TNF-α for 12 h. The expression of pri-miR-122 was analyzed by real-time PCR. **H.** HepG2 cells were transfected with miR-122 mimic or control mimic (left) and Huh-7 cells were transfected with miR-122 inhibitor or control inhibitor (right). miR-122 levels were assessed 48 h after transfection by real-time PCR. Data are presented as the mean ± SD from three independent experiments. *p*<0.05 (*), *p*< 0.01 (**), and *p*<0.0001 (***), compared to controls.

To determine whether miR-122 expression is affected by chronic hepatitis, we measured miR-122 levels in liver tissues by real-time PCR. As seen in Figure 1A, miR-122 levels were lower in CHB and HCC patients compared to healthy controls (CHB *vs.* healthy controls, 0.47±0.15 *vs.* 1.00±0.072, *p*<0.05; HCC *vs.* healthy controls, 0.20±0.029 *vs.* 1.00±0.072, *p*<0.01) (Figure 1A). As chronic inflammation may elevate serum alanine aminotransferase (ALT) levels in CHB, we further examined the correlation between ALT and miR-122 expression in CHB patients. miR-122 levels were much higher in patients with low ALT levels (normal range: 10-40 U/L) than in patients with higher ALT levels (0.84±0.072 *vs.* 0.58±0.080, *p*<0.05) (Figure 1B). Notably, miR-122 levels negatively correlated with ALT levels in CHB patients (*p*<0.05) (Figure 1C).

We then performed a meta-analysis of published miR-122 target gene expression microarray data from chronic hepatitis patients (Figure 1D). The comparison of hepatitis *vs.* healthy liver samples from an array dataset [19] revealed that miR-122 target expression increased in chronic hepatitis; on the contrary, the expression of targets of miR-33a, another highly expressed liver mRNA, did not change.

A panel of cytokines implicated in chronic liver hepatitis, including IL-1α, IL-1β, IL6, TNF-α, TGF-β, IFN-γ, and IFN-α [11, 13], was screened to assess their effects on miR-122 expression. As shown in Figure 1E, treating Huh-7 cells that constitutively express miR-122 with IL-6 or TNF-α decreased miR-122 levels by 51.2% and 51.7%, respectively (*p<*0.01 for both). miR-122 was downregulated in a dose-dependent manner 4 hours after treatment with between 1 and 1,000 U/ml of IL6 or TNF-α (Figure 1F). The maximum IL6 and TNF-α concentrations in these experiments was 1,000 U/ml (20 pg/ml of IL6 or 33 pg/ml of TNF-α), which is comparable to serum levels in CHB patients (11-112 pg/ml of IL6 and 11-170 pg/ml of TNF-α) [20–22]. Moreover, similar changes in miR-122 primary transcript (pri-miR-122) levels were observed after IL6 or TNF-α treatment (Figure 1G), indicating that these cytokines downregulate miR-122 at the transcriptional level. As IL-6 and TNF-α are major inflammatory factors in chronic hepatitis, these results suggest that chronic inflammation may contribute to miR-122 downregulation in CHB.

Given that an miR-122 target, Ccl2, may induce the production of IL-6 and TNF-α by lymphocytes in miR-122 KO mice [6], we further determined whether miR-122 regulates Ccl2 expression in hepatocytes. As shown in Figure 1H, miR-122 mimic-transfected HepG2 cells displayed decreased Ccl2 expression, whereas the inhibition of endogenous miR-122 in Huh-7 cells increased Ccl2 expression. Together, these results suggest a positive feedback loop between pro-inflammatory cytokines and miR-122 in chronic hepatitis.

### Inflammatory cytokine-induced C/EBPα downregulation and c-myc-mediated C/EBPα inhibition suppresses miR-122

**Figure 2:**
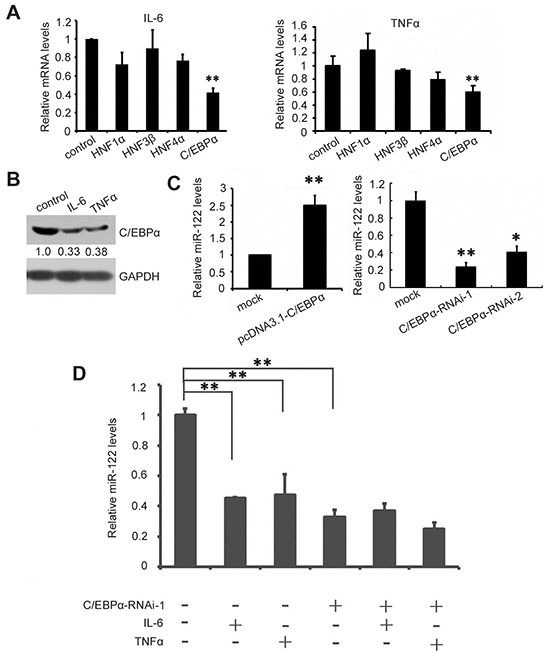
IL-6 and TNF-α suppress miR-122 by downregulating C/EBPα **A.** Huh7 cells were treated with 1000 IU/ml IL-6 (left) or TNF-α (right) or PBS as a control for 12 h, and miR-122 transcription factor mRNA levels were measured by real-time PCR (A). **B.** C/EBPα levels in Huh7 cells were analyzed by western blotting at 48 h after treatment with IL-6, TNF-α, or PBS. **C.** Huh-7 cells were transfected with pcDNA3.1-C/EBPα or pcDNA3.1 as a control (left) or with C/EBPα siRNA or control siRNA (right). miR-122 levels were assessed 48 h after transfection by real-time PCR. **D.** Huh-7 cells were transfected with C/EBPα siRNA or control siRNA. 24 h after transfection, cells were treated with 1000 IU/ml IL-6 or TNF-α for 12 h. Then, miR-122 levels were measured by real-time PCR. Data are presented as the mean ± SD from three independent experiments. *p*<0.05 (*) and *p*<0.01 (**) compared to the control.

Next, we explored the mechanism of IL-6- and TNF-α-induced miR-122 downregulation. The liver-enriched transcription factors HNF1α, HNF4α, HNF3β, and C/EBPα were examined first as these cytokines may influence miR-122 transcription [23, 24]. As shown in Figure 2A, both IL-6 and TNF-α decreased C/EBPα, HNF3β, and HNF4α mRNA levels. Of these, C/EBPα levels decreased most dramatically, and western blotting confirmed this effect (Figure 2B). C/EBPα overexpression increased, whereas RNAi-induced C/EBPα knockdown decreased, miR-122 levels (Figure 2C). Moreover, IL-6- and TNF-α-induced decreases in miR-122 expression were largely abolished by simultaneous transfection with C/EBPα siRNA (Figure 2D), indicating that IL-6 and TNF-α suppress miR-122 expression mainly by decreasing the levels of its transcription factor C/EBPα.

**Figure 3:**
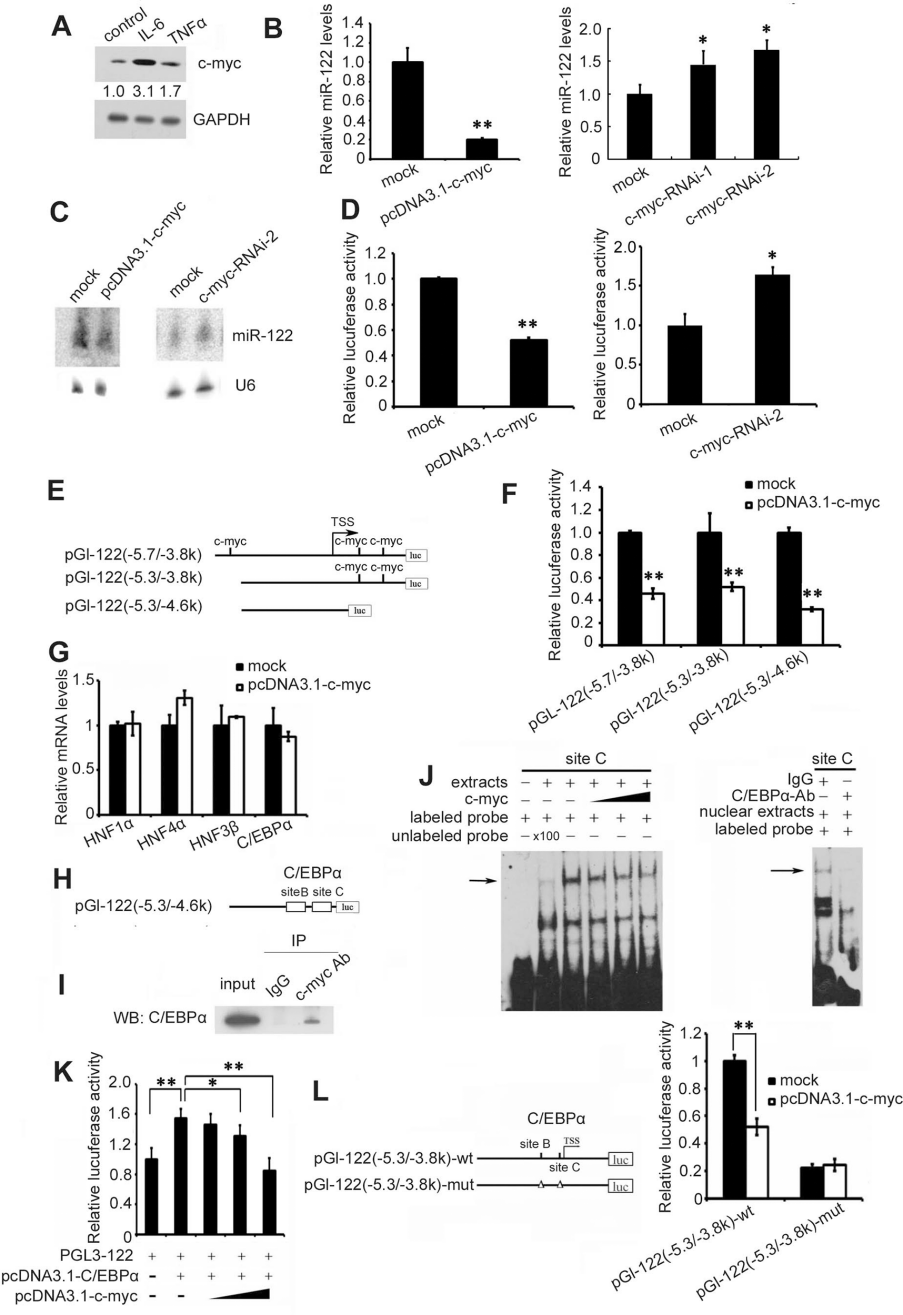
IL-6 and TNF-α suppress miR-122 by c-myc-mediated C/EBPα inhibition **A.** Huh7 cells were treated with 1000 IU/ml of IL-6 or TNF-α for 48 h, and c-myc levels were analyzed by western blotting. **B.** and **C.** Huh-7 cells were transfected with pcDNA3.1-c-myc or pcDNA3.1 as a control or with c-myc siRNA or control siRNA. miR-122 levels were assessed 48 h after transfection by real-time PCR (B) and northern blotting (C). **D.** Huh-7 cells were cotransfected with pc3.1-c-myc or pcDNA3.1 as a control or with c-myc siRNA or control siRNA, as well as with pGL-122 with a luciferase reporter under the miR-122 promoter, and pRL-TK. pGL-122 and renilla luciferase activities were measured using a dual-luciferase assay kit 48 h after transfection. pGL-122 luciferase activity was normalized to renilla luciferase activity. **E.** The miR-122 promoter pGL-122 contained three predicted c-myc binding sites (−5.7/−3.8k). Two truncated miR-122 promoter fragments, pGL-122 (−5.3/−3.8k) and pGL-122 (−5.3/−4.6k), were constructed. **F.** Huh-7 cells were cotransfected with pcDNA3.1-c-myc or pcDNA3.1 as a control, as well as pGL-122 (−5.7/−3.8k), pGL-122 (−5.3/−3.8k), or pGL-122 (−5.3/−4.6k) and pRL-TK. pGL-122 and renilla luciferase activities were measured 48 h after transfection. **G.** Huh7 cells were transfected with pcDNA3.1-c-myc or pcDNA3.1 as a control or c-myc siRNA or control siRNA. mRNA levels of HNF1α, HNF4α, HNF3β, and C/EBPα were measured 48 h after transfection by real-time PCR. **H.** Two verified C/EBPα binding sites exist in pGL-122 (−5.3/−4.6k). **I.** Co-immunoprecipitation of c-myc and C/EBPα in Huh7 cells transfected with pcDNA1.3-C/EBPα. **J.** Electrophoretic mobility shift assay performed with Huh7 cell extracts and the biotin-labeled oligonucleotide probe containing the C/EBPα-binding sequence from the miR-122 promoter. The specificity of C/EBPα binding was demonstrated by depleting C/EBPα from Huh-7 extracts with C/EBPα antibody. **K.** Huh7 cells were cotransfected with pcDNA3.1-C/EBPα or the empty pcDNA3.1 as a control, as well as with increasing amounts of pcDNA3.1-c-myc, pGL-122, and pRL-TK. pGL-122 and renilla luciferase activities were measured 48 h after transfection. **L.** (Left)The pGl-122(−5.3/−3.8k)-wt construct contains two verified C/EBPα binding sites, B and C. The mutated construct pGl-122(−5.3/−3.8k)-mut was made by deleting these C/EBPα binding sites. Deletion of the C/EBPa binding sites is depicted as a triangle (Δ). (Right) Huh-7 cells were cotransfected with pcDNA3.1-c-myc or pcDNA3.1 as a control and wild-type or mutated pGL-122(−5.3/−3.8k), and pRL-TK. pGL-122 and renilla luciferase activities were measured 48 h after transfection. Data are presented as the mean ± SD from three independent experiments. *p*<0.05 (*) and *p*<0.01 (**) compared to the control.

Given that IL-6 and TNF-α upregulate c-myc expression [25, 26] and that c-myc and miR-122 reciprocally regulate each other in HCC [27], we further investigated whether IL-6 and TNF-α also affect miR-122 expression via c-myc. As in previous studies, IL-6 and TNF-α increased c-myc expression (Figure 3A), and overexpression and knockdown studies both showed that c-myc suppressed miR-122 expression as measured by real-time PCR (Figure 3B) and northern blotting (Figure 3C). Moreover, c-myc inhibited miR-122 promoter activity in luciferase reporter assays (Figure 3D), indicating that c-myc affects miR-122 expression at the transcriptional level. Three predicted c-myc binding sites were found in the miR-122 promoter region (-5565nt to -4186 nt) using TESS analysis (http://www.cbrc.jp/research/db/TFSEARCH.html) (Figure 3E and Supplementary Figure S1). However, activity of full-length and truncated miR-122 promoter fragments with no c-myc binding sequences was similarly inhibited in pcDNA3.1-c-myc plasmid-transfected cells (Figure 3F). Similar results were observed with a miR-122 promoter containing mutated c-myc binding sites (data not shown), indicating that the negative regulation of miR-122 by c-myc is independent of its binding to the miR-122 promoter.

Next, we investigated the mechanisms underlying c-myc-mediated miR-122 downregulation. As shown in Figure 3G, c-myc did not influence the expression of miR-122 transcription factors. Because the truncated promoter fragments without c-myc binding sequences contained two verified C/EBPα binding sites [28] (Figure 3H and Supplementary Figure S1) and c-myc may bind to and inactivate C/EBPα [29], co-immunoprecipitation for these proteins was then performed in Huh7 cells. As shown in Figure 3I, endogenous c-myc bound to C/EBPα. EMSA experiments were then performed using C/EBPα-transfected Huh-7 cell extracts and a biotin-labeled oligonucleotide probe containing the C/EBPα-binding sequence from the miR-122 promoter. Competition with purified His-tagged c-myc protein expressed in *Escherichia coli* showed that c-myc inhibited C/EBPα binding to the miR-122 promoter in a dose-dependent manner (Figure 3J, left), indicating that c-myc binds to C/EBPα and blocks its association with the miR-122 promoter. The intensity of the radiolabeled DNA-protein complexes was markedly reduced when excess (100x) unlabeled oligonucleotide probe was added. The specificity of C/EBPα binding to the miR-122 promoter was confirmed by depleting C/EBPα in Huh-7 extracts with a C/EBPα antibody (Figure 3J right). Furthermore, increased c-myc levels reduced C/EBPα-mediated activation of the miR-122 promoter (Figure 3K), and c-myc suppressed the activity of the wild type miR-122 promoter but not a promoter with mutations in the two C/EBPα binding sites (Figure 3L).

Taken together, these results suggest that IL-6 and TNF-α decrease miR-122 expression directly by downregulating its transcription factors C/EBPα and HNF3β and indirectly by upregulating c-myc, which blocks the association of C/EBPα with the miR-122 promoter.

### Decreased miR-122 is correlated with IL-6 and TNFα induction in diethylnitrosamine (DEN)-induced inflammation and HCC in mice and rats

**Figure 4:**
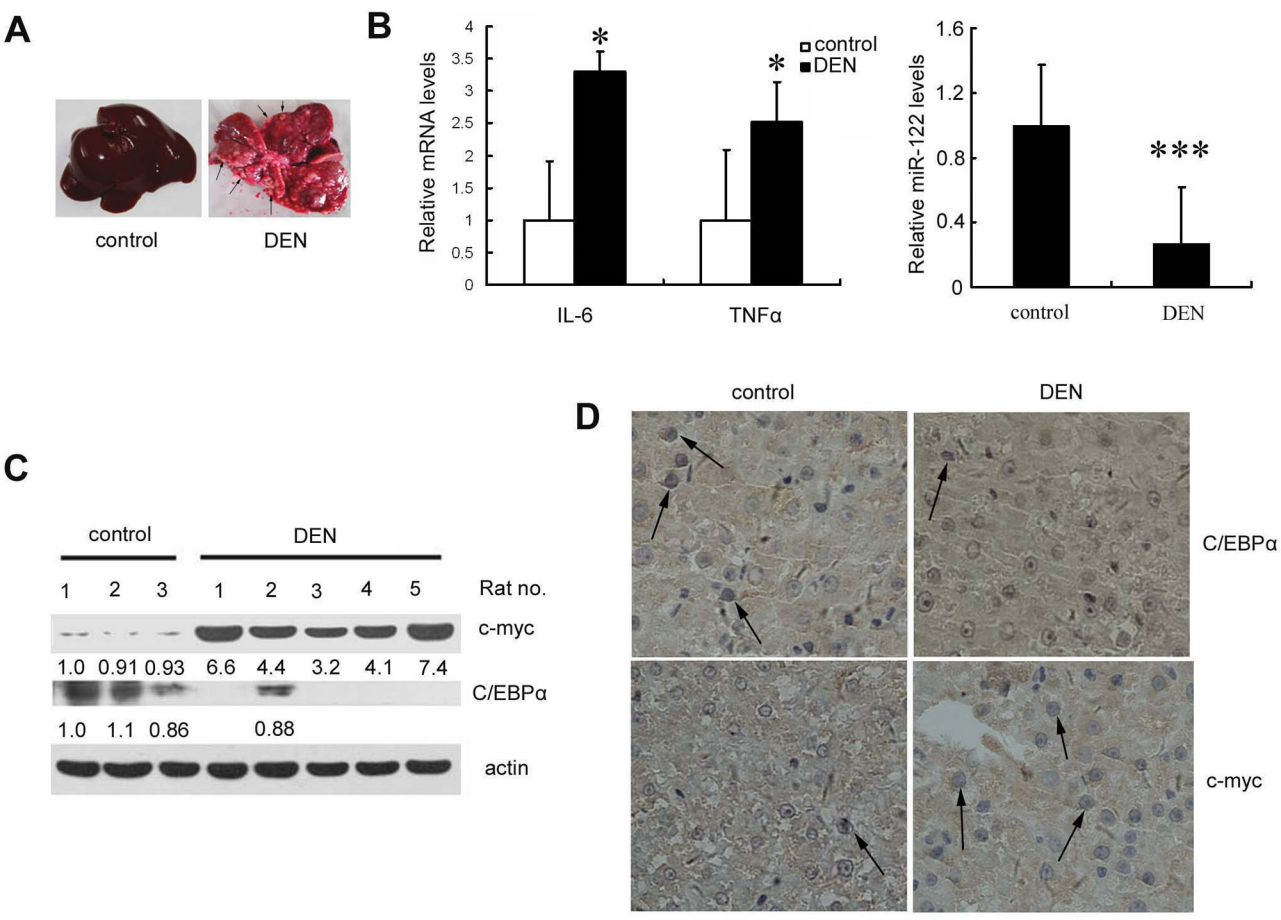
Liver miR-122 expression is decreased, whereas IL-6 and TNF-α expression are increased, in a DEN-induced rat hepatoma model **A.** Livers from DEN- or PBS (control)-treated rats are shown. Pathogen-free male Sprague-Dawley rats (weighing 160-180g) were given weekly intraperitoneal (i.p.) injections of DEN at 70 mg/kg or PBS for 10 weeks. Each group contained at least five rats. Ten weeks after the last injection, rats were sacrificed and livers were immediately removed and photographed. Arrows indicate tumors. **B.** Real-time PCR detection of IL-6 and TNF-α mRNA levels (left) and miR-122 levels (right) in liver tissues. **C.** and **D.** Analysis of c-myc and C/EBPα levels in rat livers by western blotting (C) or IHC (D). *p*<0.05 (*) and *p*<0.001 (***) compared to the control.

In the rat model of hepatocarcinogenesis, DEN induces toxic hepatitis, subsequent fibrosis or cirrhosis, and eventually the development of HCC [16]. As shown in Figure 4A, chronically exposing rats to DEN caused HCC after approximately 20 weeks of treatment. Compared to untreated rats, DEN-treated rats had higher IL-6 and TNF-α expression and lower miR-122 levels in liver tissues after 20 weeks of treatment (Figure 4B). Furthermore, immunoblotting and immunostaining confirmed that C/EBPα expression was reduced and c-myc expression was increased in DEN-treated rats compared to untreated rats (Figure 4C and 4D).

**Figure 5:**
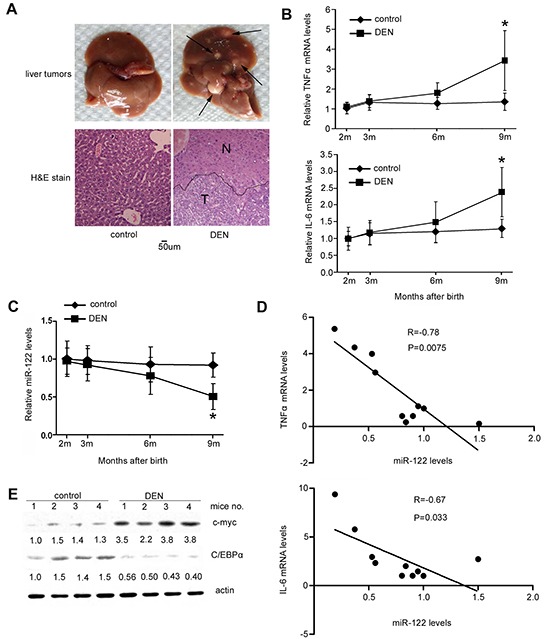
Decreased miR-122 is correlated with IL-6 and TNF-α induction in a DEN-induced mouse HCC model **A.** Fifteen days after birth, C57BL/J male mice were intraperitoneally injected with DEN at 25 mg/kg or PBS as a control. Each group contained at least five mice. Mice were sacrificed 8.5 months after injection and livers were excised and photographed (top). Arrows indicate tumors. (Bottom) HE staining of livers from DEN-injected mice and control mice. The black line shows the edges of the normal liver area (N) and tumor area (T). **B.** and **C.** Curves showing changes in TNF-α mRNA (left), IL-6 mRNA (right) (B), and miR-122 (C) levels over time in mouse liver tissues after DEN injection. **D.** Correlations between miR-122 and TNF-α or IL-6 mRNA levels in all mouse livers by Spearman analysis. Correlation coefficient values (R) are shown. **E.** Analysis of c-myc and C/EBPα levels in mouse livers by western blotting. *p*<0.05 (*) compared to the control.

Similarly, the DEN-induced autochthonous hepatocarcinogenesis model in mice mirrors inflammation-induced HCC development, in which elevated IL-6 and TNF-α levels play important roles [11]. Male mice were intraperitoneally injected with DEN 15 days after birth, and HCC development began when they were 8 months old; all mice had developed HCC by around month 9 after DEN administration (Figure 5A). Expression of IL-6 and TNF-α in the liver increased 3-6 months after DEN treatment (Figure 5B), and miR-122 levels simultaneously decreased (Figure 5C). Spearman analysis revealed a negative correlation between miR-122 levels and IL-6 (r=−0.67, *p*<0.05) and TNF-α (r=−0.78, *p*<0.01) levels (Figure 5D). As in the rat model, western blotting analysis of liver tissues showed that C/EBPα levels decreased and c-myc expression increased at month 9 in DEN-treated mice compared to untreated mice (Figure 5E).

### Agomir-122 restores miR-122 levels and suppresses DEN-induced hepatocarcinogenesis

**Figure 6:**
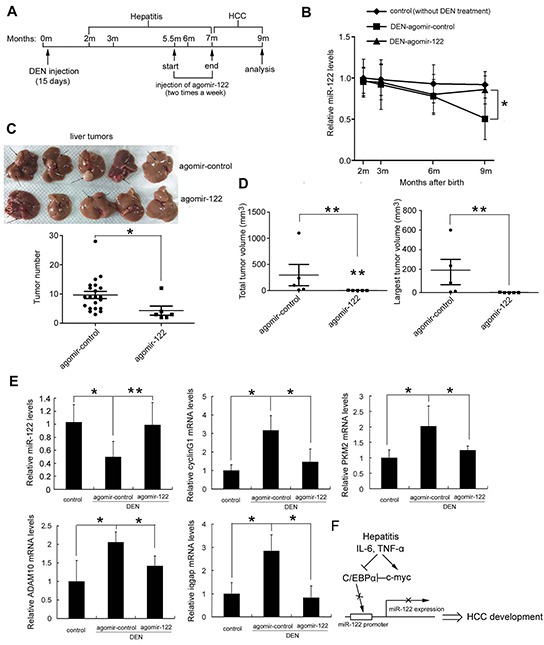
Restoration of miR-122 levels by agomir-122 delivery suppressed DEN-induced hepatocarcinogenesis Five and a half months after DEN injection, mice were randomly divided into two groups (5/group) and were intravenously injected with 5 nmol agomir-miR-122 or agomir control. Two months after injection, all mice were sacrificed, and livers were excised. **A.** Schematic diagram of agomir-miR-122 treatment in mice. **B.** Curve showing changes in miR-122 levels over time in mouse liver tissues determined by real-time PCR. **C.** Livers (top) and tumor numbers in the livers (bottom) of agomir-122 and agomir control treated mice are shown. **D.** Total tumor volume and the largest tumor volume. **E.** Real-time analysis of miR-122 levels and levels of the miR-122 target genes in liver tissues. These experiments were repeated at least twice with comparable results. **F.** Schematic figure showing how IL-6/TNF-α- C/EBPα-miR-122 may mediate inflammation and HCC development in hepatitis. Stimulation (↑) or inhibition (┬) is illustrated. *p*<0.05 (*) and *p*<0.01 (**).

To assess whether miR-122 downregulation is involved in DEN-induced hepatocarcinogenesis, we utilized a cholesterylated stable miR-122 mimic with two oxygen methylation modifications and sulfur-modified phosphate, agomir-122, to deliver miR-122 to mouse livers. Mice were treated with DEN 15 days after birth, and agomir-122 or control agomir [30] was intravenously injected 20 times at a dose of 5 nmol/mouse after 5 months (Figure 6A), when miR-122 was significantly downregulated but microscopic tumors had not yet developed. As shown in Figure 6B, treatment with agomir-122 largely restored miR-122 levels in DEN-treated mice; their miR-122 levels were similar to those in untreated control mice. At 9 months of age, mice were sacrificed and tumor burden was assessed. Compared to control mice, the total number of tumors and tumor size were strongly reduced in agomiR-122 mice (all *p*<0.05 or 0.01) (Figure 6C and 6D). As expected, the restoration of miR-122 in DEN-treated mice blocked upregulation of most of the miR-122 target genes implicated in hepatocarcinogenesis, including cyclin G1, PKM2, ADAM10, and iqgap (Figure 6E). These results indicate that miR-122 downregulation contributes to inflammation-induced HCC.

## DISCUSSION

Sustained inflammation is an important risk factor for HCC in CHB. The biological and clinical significance of miR-122 in HCC development has been investigated in numerous mouse models and clinical analyses [31]. Despite the key role of miR-122 in HCC pathogenesis, very little is known regarding its functional relevance in inflammation-mediated HCC. In this study, we demonstrated that major proinflammatory cytokines reduced miR-122 expression, which contributed to HCC development. MiR-122 levels were reduced in both CHB and HCC patients, and this decrease may be associated with chronic inflammation. Two major inflammatory cytokines in CHB, IL-6 and TNF-α, suppressed miR-122 expression both by downregulating the miR-122 transcription factors C/EBPα and HNF3β and by inducing c-myc-mediated C/EBPα inhibition. Importantly, decreased miR-122 expression enhanced proinflammatory chemokine Ccl2 expression. Furthermore, elevated IL-6 and TNF-α and decreased C/EBPα and miR-122 levels were also observed in DEN-induced rat and mouse HCC models. Notably, blocking DEN-induced miR-122 downregulation with agomiR-122 treatment significantly attenuated HCC development in mice. Our findings demonstrate that inflammatory IL-6 and TNF-α suppress miR-122 by both directly downregulating C/EBPα and indirectly reducing its transcriptional activity, as shown in Figure 6F. In this model, miR-122 acts as an HCC suppressor, and inflammation-induced decreases in miR-122 levels contribute to hepatocarcinogenesis. Dramatic decreases in miR-122 levels are observed in patients with chronic HBV or HCV infections [18, 32]. Several studies indicate that HBV X protein suppresses miR-122 transcription via binding to PPARγ, and HBV and HCV RNA/mRNAs with miR-122 binding sites sequester endogenous miR-122, which decreases its levels [10, 33, 34, 35]. Thus, both chronic inflammation and viral replication (*e.g*., transcripts) may be involved in miR-122 downregulation in the liver by HBV or HCV infection.

In this study, we identified an inflammation-C/EBPα-miR-122 regulatory loop in chronic hepatic inflammation. IL-6 and TNF-α suppressed miR-122 expression by inhibiting C/EBPα expression and transcriptional activity (Figure 2 and 3), and decreased miR-122 may result in elevated Ccl2 (Figure 1H). A previous study suggested that IL-6 represses C/EBPα transcription by inhibiting autoactivation of its promoter [36]. A similar effect was observed for TNF-α [37].

Several lines of evidence show that decreased miR-122 may be associated with inflammation. Downregulation of miR-122 results in ADAM17 upregulation, which leads to increased TNF-α production [38]. Furthermore, in miR-122 KO mice, upregulation of the miR-122 target Ccl2 results in liver inflammation and hepatic infiltration of IL-6- and TNF-α-producing inflammatory cells [6]. Together with these studies, our current work suggests that proinflammatory cytokines inhibit the transcription factor C/EBPα by repressing both its autoregulation and its transcriptional activity via c-myc, ultimately leading to decreased miR-122 expression in chronic hepatitis. In turn, miR-122 downregulation promotes further inflammation both directly by increasing inflammatory cytokine expression and indirectly by recruiting inflammatory cells in the liver. This positive miR-122 feedback loop may initiate a well-described pathogenic sequence of persistent inflammation that would predispose patients to HCC [39].

In rat and mouse DEN-induced HCC models, elevated IL-6 and TNFα expression and reduced miR-122 expression were observed in the liver before HCC development. Expression of miR-122 target oncogenes, namely cyclin G1, ADAM10, PKM2, and iqgap, was also increased in the tumors that developed (Figure 6E). Importantly, miR-122 restoration effectively reduced inflammation-mediated HCC incidence, suggesting that miR-122 may be a useful therapy in chronic hepatitis. Furthermore, delivery of miR-122 using agomir suppressed HCC development in the context of chronic hepatitis. In contrast to its supportive role in HCV, miR-122 reduces HBV expression and replication [18, 31], suggesting that miR-122 treatment might be most effective for HCC arising in the context of HBV infection. Additionally, miR-122 suppresses Ccl2 expression in hepatocytes, which actively modulate immune responses and inflammation locally in the liver [40]. The ability of miR-122 treatment to reduce inflammation in chronic hepatitis is worthy of further investigation.

In conclusion, this study indicates that chronic inflammation reduces miR-122 expression through C/EBPα in hepatocytes during chronic hepatitis, and miR-122 downregulation in turn promotes inflammation. This C/EBPα-miR-122 inflammatory feedback circuit may contribute to maintaining an inflammatory microenvironment and promote inflammation-driven HCC. Our results also suggest that the restoration of miR-122 levels may be a novel therapeutic approach for preventing HCC development in patients with chronic hepatitis.

## MATERIALS AND METHODS

### Patients and human specimens

Liver specimens from 19 CHB and 22 HBV-infected HCC patients were collected for miR-122 analysis. CHB was diagnosed according to the 2006 Diagnostic and Treatment Guidelines for Liver Failure issued by the Chinese Society of Infectious Diseases and Chinese Society of Hepatology, Chinese Medical Association. Briefly, CHB patients were defined as those with chronic HBV infection and detectable serum HBsAg levels for more than 6 months; CHB patients may also have shown hepatitis symptoms and abnormal hepatic function. The CHB patients were divided into two groups with respect to alanine aminotransferase (ALT) levels (ALT < 40, n=11; ALT ≥ 40, n=9). The standard for diagnosis of HCC was described previously (10). In addition, the unused portions of 11 normal donor livers were collected during liver transplantation to serve as controls. All patients were hospitalized in Beijing You'an Hospital of Capital University of Medical Sciences between August 2010 and August 2012. All study participants provided written informed consent. Patient samples were assigned arbitrary identification numbers based on the order of enrollment in our study. The study protocol was approved by the Ethics Committee of Beijing You'an Hospital.

### Reagents and antibodies

Chemically synthesized c-myc- and C/EBPα-specific small interfering RNA (siRNA) and nonspecific control were purchased from RiboBio Co., Ltd. (Guangzhou, China). The sequences of the c-myc siRNAs were 5′-CTATGACCTCGACTACGAC-3′ and 5′-CTCGGTGCAGCCGTATTTCTA-3′, and the C/EBPα siRNA sequences were 5′-CCAAGAA GUCGGUGGACAADTDT-3′ and 5′-CGACGAGTTC CTGGCCGAC-3′. We purchased cholesterol-conjugated miR-122 mimic and negative control from Ribobio (Guangzhou, China) for RNA delivery in vivo. Reagents and antibodies were obtained as follows: hIL-6 (recombinant human Interleukin 6) and hTNFα (recombinant human tumor necrosis factor α) were purchased from Sinobio Biotechnology Co., Ltd. Shanghai China; DNase I (RNase-free) was purchased from Invitrogen; the Superscript RT reagent kit was from TaKaRa Bio Inc., Shiga, Japan; rabbit anti-human C/EBPα (sc-61 X) and mouse anti-c-myc (sc-40) monoclonal antibodies were from Santa Cruz Biotechnology; rabbit IgG was from Sigma; mouse anti-human actin, GAPDH and horseradish peroxidase (HRP)-conjugated secondary antibodies were purchased from Zhongshan Goldenbridge Biotechnology, China; the ECL-Plus chemiluminescence system was from Applygen Technologies, Beijing, China.

### Plasmid constructs

The human c-myc gene was cloned into pcDNA3.1 or pET28a vectors named pCDNA3.1-c-myc or pET28a-c-myc, respectively. pHBV containing 1.3 copies of the HBV genome (D genotype) and the HBV replication plasmid were maintained in the lab. The HBV-luciferase plasmid (pHBV-Luc), containing a luciferase open reading frame (ORF) under the control of HBV enhancers and the core promoter, was kindly provided by Yosef Shaul (The Weizmann Institute of Science, Israel). The construct pGL-122 (−5.7/−3.8k), containing a luciferase reporter gene under the control of the miR-122 promoter, and pCDNA3.1-C/EBPα was kindly provided by Shi-Mei Zhuang (Sun Yat-Sen University, China). The pGl-122 construct containing two verified C/EBPα binding sites, B and C, was used to create the pGl-122-mut construct with deleted C/EBPα binding sites. The point mutated primers were synthesized and used in Polymerase Chain Reaction (PCR). To verify three predicted c-myc binding sites on the miR-122 promoter, pGl-122 (−5.3/−3.8k) containing 2 c-myc binding sites and pGl-122 (−5.3/−4.6k) containing no binding sites were constructed using *Kpn*I & *Xho*I double digestion.

### RNA extraction and real-time PCR

Total RNA was extracted using TRIzol reagent (Invitrogen, Carlsbad, CA, USA). Real-time PCR analysis for miR-122 was performed using a TaqMan miRNA kit (Applied Biosystems, Foster City, CA, USA). The U6 endogenous control was used for normalization. miR-122 primary transcript (pri-miR-122) was detected using a high-capacity cDNA reverse transcription kit (Applied Biosystems, Foster City, CA, USA) and a TaqMan pri-miRNA assay. Actin was used as an internal standard gene. Real-time fluorescence quantitative PCR was performed using SYBR green premix reagent (TaKaRa Bio Inc., Shiga, Japan). Glyceraldehyde-3-phosphate dehydrogenase (GAPDH) was used as an internal standard for quantification. Primers used in RT-PCR were chemically synthesized and are shown in Supplementary Table S1, S2 and S3. Relative expression was quantified using the comparative threshold cycle (CT) method.

### Electrophoretic mobility shift assay (EMSA)

The recombinant c-myc protein was expressed and purified as 6Xhis fusion protein in *E. coli* DL21. Nuclear extracts from lysed Huh7 cells were prepared using a Nuclear-Cytosol Extraction Kit (Applygen Technologies, Beijing, China). 3′-biotin-labeled complementary oligonucelotide pairs containing the C/EBPα binding sequences from the miR-122 promoter were chemically synthesized and annealed. The oligonucelotide sequences were 5′-GAGAAAGAATTGTTTACTTTTAAACCCTGGA-3′(forward) and 5′-TCCAGGGTTTAAAAGTAAACA ATTCTTTCTC-3′(reverse). Nuclear extracts (4 μg) were mixed with recombinant c-myc protein at 4°C for 1 h and then added to the binding reaction. For the antibody-supershift assay, 2 μg of anti-C/EBPα antibody or 2 μg of control rabbit IgG were preincubated with nuclear extract at 4°C for 1 h and then added to the binding reaction. The mixture was incubated with 50 fmol of the 3′-biotin-labeled oligonucleotide probe. DNA-protein complexes were loaded on a 5% non-denaturing polyacrylamide gel at 4°C and visualized by chemiluminescence.

### Cell culture and transfection

Human hepatoma cell lines Huh-7 and HepG2 were obtained from the ATCC (Manassas, VA, USA). The cell lines were authenticated by short tandem repeat DNA testing at Beijing Microread Gene Tech., Co., Ltd. in 2015. Huh-7 and HepG2 were cultured in Dulbecco's Modified Eagle's medium. Transfections were performed using Lipofectamine 2000 reagent (Invitrogen, Carlsbad, CA, USA). Cells were transfected with 50 nM siRNA or 2ug expression plasmid. Each treatment was performed at least three times.

### Luciferase reporter assays

Cells were co-transfected with pGL-122, pRL-TK plasmid, and the indicated plasmids. 48 h after transfection, cells were harvested and detected with the Dual Luciferase Reporter Assay System (Promega, Madison, WI, USA).

### Immunohistochemistry (IHC)

Liver tissue sections (4 μm thick) were immunostained using the c-myc (sc-40) and C/EBPα (sc-61X) antibodies (Santa Cruz Biotech, Dallas, Texas, USA) and a horseradish peroxidase (HRP)-conjugated secondary antibody and were visualized with 3,3′-diaminobenzidine tetrahydrochloride (DAB).

### Northern blot analyses

Northern blot assays of miR-122 were performed as described previously (10).

### Immunoprecipitation and immunoblot analysis

Huh-7 cells were transfected with the pCDNA3.1-C/EBPα plasmid and then lysed in lysis buffer with protease inhibitor (Roche Applied Science, Indianapolis, USA) at 4°C for 30 min. The cell lysates were incubated with a mouse anti-c-myc (sc-40) monoclonal antibody or a mouse control IgG at 4°C overnight, followed by the addition of protein G-sepharose beads (GE Healthcare, USA). After 2 h of incubation at 4°C, the beads were washed with lysis buffer. The immunoprecipitates were separated by SDS-PAGE and immunoblotted with the anti-flag antibody.

### Animal studies

Animals were treated according to the “Guide for the Care and Use of Laboratory Animals” by the National Academy of Sciences (NIH publications No. 80-23, revised 1996). All mice and rats were obtained from the Institute of Laboratory Animal Science (CAMS&PUMC). Fifteen days after birth, C57BL/J male mice were intraperitoneal injected with DEN at 25 mg/kg or with PBS as a control. Five months after injection, the DEN-injected mice were randomly divided into three groups (6 mice/group). Two groups of mice were intravenously injected with 5 nmol cholesterol-conjugated miR-122 (agomir-miR-122) in 0.1 ml saline buffer. Ten weeks after the last agomir-miR-122 or control agomir injection, all mice were sacrificed and the livers were excised. Externally visible tumors (≥ 1 mm) in the livers were counted and the liver tissues were stored at −80°C for RNA extraction. Pathogen-free male Sprague-Dawley rats (weighing 160-180 g) received weekly intraperitoneal (i.p.) injections of DEN at 70 mg/kg or of PBS as a control over 10 weeks. Ten weeks after the last DEN injection, rats were sacrificed after ether anesthesia, and the livers were immediately removed. The liver tissues were stored at −80°C for RNA extraction.

### Statistical analysis

Differences between groups were determined using Student's *t*-tests. The degree of association between variables was determined by Spearman's nonparametric correlation. *p*<0.05 was considered significant.

## SUPPLEMENTARY FIGURE AND TABLES



## Abbreviations

HCC: hepatocellular carcinoma
miR-122: microRNA-122
CHB: Chronic hepatitis B
DEN: diethylnitrosamine
miRNA: microRNA
KO: knockout
ADAM10: distintegrin and metalloprotease family 10
SRF: serum response factor
Igf1R: insulin-like growth factor 1 receptor
PTTG1: pituitary tumor-transforming gene 1
PKM2: Pyruvate Kinase M2
ALT: alanine aminotransferase
EMSA: Electrophoretic Mobility Shift Assay
pri-miR-122: miR-122 primary transcript
