# Supplementary material for: Chronic inflammation contributes to the development of hepatocellular carcinoma by decreasing miR-122 levels
Source: Oncotarget. 2016 Feb 26;7(13):17021–34. doi: 10.18632/oncotarget.7740 (PMC4941368; doi:10.18632/oncotarget.7740)
Supplement: Supplementary file 1 [file oncotarget-07-17021-s001.pdf]

SUPPLEMENTARY FIGURE AND TABLES

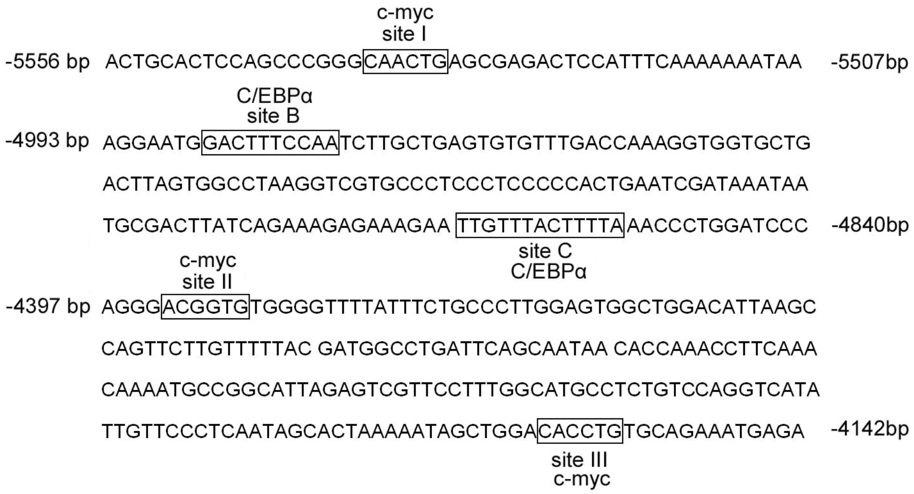

Supplementary Figure S1: Sequence analysis of the human miR-122 promoter using the TESS - Transcription Element Search System analysis. The putative binding sites for c-myc and C/EBPα are indicated.

Supplementary Table S1: Human primers for real-time PCR

HNF1a-F	CCTCAAAGAGCTGGAGAACCT
HNF1a-R	GACTTGACCATCTTCGCCAC
HNF3β-F	CGACTGGAGCAGCTACTATGC
HNF3β-R	TACGTGTTCATGCCGTTCAT
HNF4a-F	GCAGGCTCAAGAAATGCTTC
HNF4a-R	GGCTGCTGTCCTCATAGCTT
CCL2-F	GCTCAGCCAGATGCAATCA
CCL2-R	AGATCTCCTTGGCCACAATG

Supplementary Table S2: Rat primers for real-time PCR

IL-6-F	CGAGCCCACCAGGAACGAAAGTC
IL-6-R	CTGGCTGGAAGTCTCTTGCGGAG
TNFa-F	GACCCTCACACTCAGATCATCTTCT
TNFa-R	TGCTACGACGTGGGCTACG
GAPDH-F	GGCAAGTTCAACGGCACAGT
GAPDH-R	TGGTGAAGACGCCAGTAGACTC

SupplementaryTable S3: Mice primers for real-time PCR

CCNG1-F	CAGTTCTTTGGCTTTGACACG
CCNG1-R R	TTCCTCTTCAGTCGCTTTCAC
PKM2-F	CGCCTGGCGCCCATTACCAGC
PKM2-R	TTGGTGAGCACGATAATGGCCC
ADAM10-F	TGATGGTGTTCTTGGTCTGG
ADAM10-F	CTTGCTTTTCTCACATATTCCCC
IQGAP1-F	TTCTCTCCCAAAGTGGTGTCCC
IQGAP1-R	TCTTAGGCAACCCAATCTCATCC
GAPDH-F	ACTTTGGCATTGTGGAAGG
GAPDH-R	ACACATTGGGGGTAGGAACA
